# Effects on Consumers’ Subjective Understanding and Liking of Front-of-Pack Nutrition Labels: A Study on Slovenian and Dutch Consumers

**DOI:** 10.3390/foods10122958

**Published:** 2021-12-01

**Authors:** Angelo Baccelloni, Andrea Giambarresi, Marco Francesco Mazzù

**Affiliations:** 1 Department of Communication and Social Research, Sapienza University of Rome, Via Salaria, 00198 Roma, Italy; 2 Department of Business and Management, LUISS Guido Carli University, Viale Romania, 00197 Rome, Italy; agiambarresi@luiss.it; 3 Luiss Business School, Via Nomentana, 00162 Roma, Italy; mmazzu@luiss.it

**Keywords:** front-of-pack nutritional label, Nutri-Score, NutrInform Battery, subjective understanding, liking, overweight, obesity, Slovenia, Netherlands

## Abstract

In the context of the ongoing debate on front-of-pack labels (FOPL), extant research highlights a lack of clear indications on which label is most effective in increasing consumers’ knowledge of food nutritional quality, and in favoring informed food choices. In this study, we have compared the effects of two different labels, one nutrient-specific label (i.e., NutrInform Battery) and one summary label (i.e., Nutri-Score), in terms of consumers’ “subjective understanding” and “liking”. Our work advances prior research on FOPL performance by focusing on two different countries—which have different socio-political contexts and which, from previous studies, present limited evidence on the topic—Slovenia, currently utilizing the Protective Food logo, and the Netherlands, who has recently adopted the Nutri-Score. The study also confirms, in line with previous research, a higher effectiveness of the nutrient-specific label, NutrInform Battery, on all analyzed dimensions in tested countries, when compared to the summary label, Nutri-Score.

## 1. Introduction

In the last few years, the phenomena of overweight and obesity have emerged as some of the most serious global health problems, with consequences on several non-communicable diseases [1]. Their rate has nearly tripled since 1975: recent predictions forecast that 50% of global citizens will be overweight by 2030 [2]. In 2016, 1.9 billion people over 18 were considered overweight, whereas in the youngest part of the population, ranging from 5 to 19 years old, 340 million of people were found to be overweight or obese [1]. In response to this growing issue, governmental bodies (e.g., EU, WHO) have, since 2006, undertaken actions to support consumers in making informed food choices, with the goal of decreasing obesity, and its direct and indirect costs on public health systems.

The COVID-19 pandemic has worsened the situation for the obese population. The risk of being hospitalized with COVID-19, for people who are overweight or obese, is increased by 113%, the risk of being admitted to the intensive care unit by 74%, and the risk of dying by 48% [3]. Public Health England has registered similar numbers regarding mortality reporting an increase of 90% of the risk of death from COVID-19 for individuals with BMI rate greater than 40 [3]. Furthermore, a World Obesity Federation report released on 4 March 2021 showed additional trends, noting that COVID-19 mortality rates were ten times higher in countries in which more than half of the population is obese [3].

In line with the World Health Organization (WHO), these forecasts can be avoided from happening through an integrated set of actions at different levels: individual; industrial; and governmental [1].

In this context, the EU introduced specific regulations (e.g., 1169/2011) for the establishment of food front-of-pack labels (FOP labels or FOPLs) that can support consumers in making informed food choices, and the WHO [1] identified FOPLs as an effective tool to limit the increase of diet-related diseases.

As a result, numerous member states have begun to develop their own front-of-pack labels (Table 1) [4], such as the United Kingdom in 2013 (Multiple Traffic Light), France in 2017 (Nutri-Score), and Italy in 2019 (NutrInform Battery). The introduction of these FOPLs has led to a debate on which label should be adopted for the whole European Union [5]. These labels follow different—and complementary—approaches, and can be classified, according to the recent EU taxonomy (Table 1), into nutrient-specific or summary labels [6]. Nutrient-specific labels present detailed numerical data regarding the nutritional values of foods, specifically on energy value, fat, saturated fat, sugar, and salt, and also through percentages and the usage of colors and symbols. On the other hand, summary labels provide an evaluation of the general healthiness of the product, often through algorithms.

This study aims at further contributing to the European discussion on label adoption, comparing the performance of different FOPLs in two countries with limited evidence on the impact of FOPL on consumers, with specific attention to the pre-usage phase of subjective understanding and liking.

### Extant Literature and Research Objectives

Grunert and Wills [7] modelled the consumer decision-making process regarding food, and outlined the five stages that lead to the use of a product: exposure; perception; understanding; liking; and use.

Among the factors that influence the usage phase, the two different elements that form understanding—objective and subjective—have been deeply investigated by the recent FOPL literature. 

Objective understanding is defined whether the meaning attributed by the consumer to the label is in line with the meaning intended by the sender of the label (i.e., if the consumers have understood what the sender wanted to communicate), whereas subjective understanding is the meaning that the consumer obtains from the perception of the information present on the labels (i.e., what consumers believe they understand about nutrition factors relevant to their nutrition knowledge).

Recent studies (Table 2) compared the performance of different FOPLs along the two dimensions of understanding, and produced on one side, coherent results within each research stream, and on the other side, contrasting evidences and outputs between objective and subjective understanding [8]. Specifically, summary labels have been shown to be more effective in supporting objective understanding, also due to the use of color, which attracts consumers’ attention, and facilitates decision-making in time-constrained situations [9,10,11,12]. Recent research [13] performed in Slovenia confirmed the higher ability of the Nutri-Score to differentiate food products on the basis of composition. A recent study conducted in a Dutch supermarket by Van den Akker et al. [14] on FOPLs’ effectiveness in guiding consumer choices reported that the Nutri-Score can help consumers toward healthier choices, while not increasing the intended serving sizes.

Studies on subjective understanding have also shown that consumers are able to better understand nutritional values when using a nutrient-specific label, as this kind provides more complete and specific information [15,16,17].

Our research tests two labels that are at the extremes of the EU taxonomy to gain evidence on opposite approaches to FOP labelling: the Nutri-Score, as the most representative summary label at the EU level, and the recently introduced NutrInform Battery. Countries have been selected complementing research already fielded on similar topics [7,17], and because they represent very different socio-political contexts.

The Protective Food Logo was introduced in Slovenia in 1992, with the aim to support consumers in their decision-making process toward healthier choices, and to encourage a rework on food formulations [18]. The logo presents the specific nutrient criteria that a product has to achieve to be able to carry the symbol; in this way, the consumer is implicitly told that the product in question has met specific requirements [19]. The Protective Food Logo is based on the combination of food-traffic-light thresholds and regulatory criteria for certain nutritional and health claims—the symbol is supported by a specific claim, which presents the benefits of the product [18]. This symbol can now be found on about 2% of the products on the market, in a context where less than half of the Slovenian population (42%) does not pay attention to food labels [18]. A study from 2016 made by Miklavec et al. [20] on the Slovenian Protective Food Logo reported that 78% of the participants had previously seen the symbol, and 64% of them declared to be familiar with it. These results show an increase in familiarity of the symbol among Slovenian adults: the familiarity rate has increased from 40% in 2000 to 64% in 2016 [20]. This data shows an increased attention of the Slovenian population towards their food intake.

A study by Kupirovic et al. [18] focuses on the problem of scenarios presenting conflictual information: food can be considered healthy by the symbol, for example the Protective Food Logo, but unhealthy by scoring labels such as the Nutri-Score. 

Furthermore, the Nutri-Score has demonstrated to have some blind spots given the fact that some ingredients (e.g., synthetic ingredients) are not taken into account while the main message is that the product should be consumed in reasonable quantities [21]. In a cross-country study regarding consumers’ understanding of FOPLs, Gregori et al. [22] reported that front-of-pack labels enhance comprehension, understanding, and interpretation of nutritional information, enhancing consumer comprehension and comfort level with such information. Furthermore, the research concluded that FOP labels encourage food manufacturers to reformulate their products, and develop new products with a healthier composition. Other studies [23] have shown that in Slovenia, the willingness to pay for products with a front-of-pack label was higher than the average of the other European countries considered. In 2019, however, the adult population in Slovenia was 19.9% obese, and 38.2% was overweight [24], with a subsequent overall and childhood obesity risk.

The Netherlands recently adopted the Nutri-Score while continuing to analyse the underlying algorithm given the fact that it’s scores are not very consistent with Dutch dietary guidelines [25]. In 2020, 36.8% of the population was overweight, and 14.2% of the population was obese, increasing since 2002, where the obesity rate was 10.4%, and the overweight rate was 35.3% [24]. Due to the growing problem of obesity, before the Nutri-Score, the Netherlands adopted the voluntary Dutch Choices label, to indicate a product’s level of healthiness. The Choices label was developed in two different variants: (a) a green logo indicating which are the healthy choices within basic product categories; and (b) a blue logo indicating which are the healthier choices within non-basic product categories [19]. The results of research conducted by Vyth et al. [26] reported that exposure to the Dutch Choices label has increased significantly during recent years, and has received different responses according to the customer category. Older, obese respondents reported more need for the logo than younger, normal-weight individuals. Women evaluated the logo to be more attractive and credible than men. Further qualitative analysis has shown that: (a) if the government and scientific authorities were to support the logo, its credibility would improve; (b) older respondents said that, for them, the logo is needed because of health concerns; and (c) consumers who are interested in health use the logo. Vyth et al. in 2010 [27], in research on the purchasing behaviour of the Dutch population in grocery shops, reported that both participants who are careful about their weight and familiar with looking at nutrition information on food packages would chose products with the Choice label. Van Herpen et al. [28], who studied the role of familiarity in the evaluation of FOPLs in the Netherlands and in the United Kingdom, highlighted that the level of familiarity with the label influences self-reported evaluations, usage intentions, and healthful choices. Smed et al. [29], who carried out a study in the Netherlands using household purchasing data, reported a positive effect of labelling for the majority of products. Furthermore, products with the voluntary Dutch Choices label (which indicates that a product is a “healthy” choice) have increased the market share after displaying the scheme [29]. A study by Egnell et al. [30] compared different FOP labels (Health Star Rating, Multiple Traffic Light, Reference Intake, Warning Symbols), and tested their impact on the objective understanding of Dutch consumers: the Nutri-Score was found to have the best performance in helping consumers sort foods on the basis of their nutritional quality. Research conducted by Jewell [19] comparing the performance of FOP labels on the market highlighted that Dutch people were not able to differentiate their Choice Logo with the Green Endorsement Logo: moreover, not all of the manufacturers have participated in the scheme, causing potential confusion on whether the non-appearance of the logo implies product un-healthiness or not. Those results show the need for a label that had greater support from scientific and governmental authorities, and for this reason, in 2021, the Netherlands chose to adopt the summary label Nutri-Score.

In this context, the study has the goal of providing additional evidence concerning the relative effectiveness of two labels at the extreme of the EU taxonomy—NutrInform Battery and Nutri-Score—with the purpose of increasing consumers’ awareness, and helping consumers make healthier choices.

Based on results highlighted in previous research [15,16,17], we expect that, with respect to the three sub-dimensions of “subjective understanding” and to “liking”, a nutrient-specific label (NutrInform Battery) will perform better than a summary label (the Nutri-Score) in both tested countries, Slovenia and the Netherlands.

In particular, we hypothesize the following by considering the three sub-dimensions of subjective understanding (comprehensibility, help-to-shop, complexity):

**Hypothesis** **1a (H1a).** *The NutrInform Battery (vs. the Nutri-Score) has a higher impact on the labels’ comprehensibility in Slovenia.*

**Hypothesis** **1b (H1b).** *The NutrInform Battery (vs. the Nutri-Score) has a higher impact on the labels’ comprehensibility in the Netherlands.*

**Hypothesis** **1c (H1c).** *The NutrInform Battery (vs. the Nutri-Score) has a higher impact on the labels’ help to shop in Slovenia.*

**Hypothesis** **1d (H1d).** *The NutrInform Battery (vs. the Nutri-Score) has a higher impact on the labels’ help to shop in the Netherlands.*

**Hypothesis** **1e (H1e).** *The NutrInform Battery (vs. the Nutri-Score) has a higher impact on the labels’ complexity in Slovenia.*

**Hypothesis** **1f (H1f).** *The NutrInform Battery (vs. the Nutri-Score) has a higher impact on the labels’ complexity in the Netherlands.*

Furthermore, regarding liking, we have hypothesized that:

**Hypothesis** **2a (H2a).** *The NutrInform Battery (vs. the Nutri-Score) has a higher impact on the labels’ liking in Slovenia.*

**Hypothesis** **2b (H2b).** *The NutrInform Battery (vs. the Nutri-Score) has a higher impact on the labels’ liking in the Netherlands.*

## 2. Materials and Methods

As stimuli, in the present research, we utilized NutrInform Battery and Nutri-Score. The two labels were chosen because they represent different approaches to front-of-pack labelling, and have a different degree of presence in the extant literature. 

A between-subjects design has been utilized for the research, with the two different FOPL conditions serving as the basis for the study. Each condition was placed on mock packages of the same types of food categories, to investigate the impact of the two alternatives on four dependent variables, i.e., the three sub-dimensions of subjective understanding (comprehensibility, help-to-shop, and complexity), plus liking. Mock products have been used in order to avoid the effects of brand exposure that could mislead consumers [10]. Participants were randomly assigned to the different tested conditions. The questionnaire was structured on the Qualtrics platform, translated into the original language with the support of a professional translator, and delivered via Prolific Academic, an on-demand, self-service data collection that supports the recruitment of high-quality research participants. The questionnaire opened with the collection of socio-demographic information, followed by the core part, in which respondents were asked to provide an assessment of all the sub-dimensions of subjective understanding, and of liking. 

A description of the socio-demographics of each sample is provided in the table below (Table 3)

The items in each sub-dimensions (Table 4) were derived from previous research [31,32], and were consistent with recent past research on subjective understanding [15,16,17].

▪ Comprehensibility: “I feel well informed by the food label”; “This label is believable and trustworthy”; and “This label is easy to interpret”. We used a 7-point Likert scale from 1 = Completely Disagree to 7 = Completely Agree.
▪ Help-to-shop: “This label helps me understand the product composition”; “This label helps me to understand different nutritional values”; and “This label makes it easier to choose food”. We used a 7-point Likert scale from 1 = Completely Disagree to 7 = Completely Agree.
▪ Complexity: “The food label is rather extensive”; and “Using this food label to choose food is better than just relying on my own knowledge about what is in them”. We used a 7-point Likert scale from 1 = Completely Disagree to 7 = Completely Agree.
▪ Liking: “How do you evaluate the label?” Respondents expressed their opinion answering to the following scales: “bad/good”; “unfavourable/favourable”; and “negative/positive”. (1 = Bad, 7 = Good; 1 = Unfavorable, 7= Favorable; 1 = Negative, 7 = Positive).
  It is important to notice that the item utilised to measure the third subdimension of subjective understanding, complexity, does measure a reduction of the perceived complexity by the consumer.

We utilized three covariates—age, education, and income level—in line with past research. Extant studies highlight that lower income levels lead to a preference for evaluative/directive labels, and to lower label use [9,33,34]. The level of education influences the understanding of the label, with evidences of the fact that nutrient-specific labels have lower levels of understanding for respondents with a lower level of education [11,14,34,35]. Finally, prior studies showed how age impacts the dependent variables, with children having a preference for evaluative labels [35], whereas older people are less likely to collect additional information from FOP labels at all [36].

## 3. Results

We compared participants’ evaluations of the different conditions using Students’ *t*-tests. IBM SPSS Statistics 25 software (Armonk, NY, USA) was used to perform the analyses, only on data from participants who had completed the questionnaire appropriately, and that had passed the attention check.

We first assessed reliability by calculating the Cronbach’s alpha registered in both countries by the tested items (Table 5), and then we calculated the mean of the items for the dimensions of comprehensibility, help-to-shop, complexity, and liking.

The results reported a similar performance in both in Slovenia and the Netherlands (Table 6). The product with the NutrInform Battery registered a better performance than the one with the Nutri-Score in all the three sub-dimensions of subjective understanding, comprehensibility, help-to-shop, and complexity. Regarding liking, we registered a different performance of the two FOPLs: though in Slovenia, the NutrInform Battery significantly outscored the Nutri-Score, in the Netherlands, the differences between the means reported by the two labels were not statistically significant.

In the following part, we report in detail the effects on each tested item.

### 3.1. Subjective Understanding

#### 3.1.1. Comprehensibility

In Slovenia, NutrInform Battery reported a mean of 4.92, whereas the Nutri-Score registered a mean of 3.27, showing a statistically significant difference (t(153)= −7.46: *p* < 0.001). Similar results were seen in the Netherlands, where NutrInform Battery obtained a mean of 4.98 vs. the 4.31 of Nutri-Score, and there was a significant overperformance (t(248)= −3.80: *p* < 0.001). In both countries, NutrInform Battery performed significantly better than the Nutri-Score, confirming the results of prior research in other European countries, hence we confirm H1a and H1b.

#### 3.1.2. Help-to-Shop

Starting from Slovenia, our results reported a higher performance for the NutrInform Battery compared to the Nutri-Score regarding help-to-shop (M*_NutrInform Battery_* = 5.00; M*_Nutri-Score_* = 3.37; t(153) = −7.21, *p* < 0.001). Results were also confirmed in the Netherlands (M*_NutrInform Battery_* = 4.41 vs. M*_Nutri-Score_* = 2.75, with t(248) = −8.86, *p* < 0.001). Also, for this dimension, NutrInform Battery outperformed Nutri-Score, confirming the results obtained in other European countries, therefore we confirmed H1c and H1d.

#### 3.1.3. Complexity

The third sub-dimension of subjective understanding reported a similar performance to the previous one in both countries, and was consistent with the findings of the extant research.

In Slovenia, we reported, for the NutrInform Battery, a mean of M*_NutrInform Battery_* = 3.99, higher than the one registered for the Nutri-Score label (M*_Nutri-Score_* = 2.52), with a significant difference (t(153) = −6.07, *p* < 0.001). Furthermore, in the Netherlands, we reported a similar situation in which the NutrInform Battery showed a better performance than the Nutri-Score (M*_NutrInform Battery_* = 3.59 vs. M*_Nutri-Score_* = 2.43, t(248) = −6.43, *p* < 0.001), therefore, we confirmed H1e and H1f.

#### 3.1.4. Liking

Regarding liking, we have registered different performances of the two labels in the tested countries. In Slovenia, the NutrInform Battery obtained a significantly better performance than the Nutri-Score (M*_NutrInform Battery_* = 4.96 vs. M*_Nutri-Score_* = 4.50 with t(153) = −2.61: *p* = 0.01) in terms of consumer liking, hence, we confirmed H2a. On the other hand, in the Netherlands, we did not report a significant difference between the performances of the two labels (M*_NutrInform Battery_* = 4.32 vs. M*_Nutri-Score_* = 4.41, t(248) = −4.17, *p* > 0.05), therefore, we rejected H2b.

The results obtained in the two tested countries reported in Table 6 show a similar performance of the NutrInform Battery on comprehensibility, help to shop and complexity. In addition, the comparison between the two countries showed a higher mean for the Nutri-Score in the Slovenian population than the Dutch people, even though the Netherlands had decided to adopt it as the official label. 

## 4. Discussion and Conclusions

Our work aimed at providing additional evidence to the European debate on the harmonization of front-of-pack labels across countries, by expanding the research on the effects of two different FOPLs on consumers’ subjective understanding and liking in two nations with different attitudes toward FOPLs, in terms of different healthiness and socio-political context. Specifically, Slovenia has a non-mandatory Protective Food Logo, currently employed in a very small number of cases; the Netherlands, after having utilized the Dutch Choice Label for many years, recently adopted the Nutri-Score as the official FOPL for the country.

Our research explored consumers’ subjective understanding and liking of nutrient-specific (NutrInform Battery) and summary labels (Nutri-Score), confirming and validating the results of previous studies [15,16,17] performed in eight European countries: France; Germany; Greece; Italy; Poland; Portugal; Romania; and Spain. 

Our results showed a significant mean difference between the two labels on most of the dependent variables, both in Slovenia and in the Netherlands. Specifically, the NutrInform Battery registered a better performance vs. the Nutri-Score in all the three sub-dimensions of subjective understanding—comprehensibility, help-to-shop, and complexity—in both countries. On the other hand, regarding liking, we obtained two different results. Though in Slovenia, we reported a statistically higher mean from the NutrInform Battery, in the Netherlands, the mean difference between the two labels was not statistically significant.

The main contributions of our research to the current ongoing and unsolved debate on which FOPL might better support European consumers toward informed food choices, is the validation and reinforcement of previous results on the relative performance of Nutri-Score and NutrInform Battery in terms of subjective understanding and liking. This paper presents insights that can help European policy-makers define the type of FOPL to be adopted EU-wide. 

Our work is not exempt from limitations; for example, more countries could be analysed in order to extend the generalization of the reported results; the study focused on subjective understanding, however, further research could test the NutrInform Battery on objective understanding as well. Furthermore, future research could explore the reason of the different performance of Nutri-Score in terms of subjective and objective understanding. Also, future streams might focus on more behavioural variables. In addition, to promote informed and balanced diets among consumers, it could be useful to test supporting tools that could compute the total of calories and nutrients taken throughout the day, to track energy and nutrients in food against the recommended daily intakes.

## Figures and Tables

**Table 1:** European taxonomy on front-of-pack labels and FOPL definition by WHO.

Front of Pack Label-Definiton		
Front-of-pack labelling (FOPL) refers to nutrition labelling systems that: a) are presented on the front of food packages (in the principal field of vision) and can be applied across the packaged retail food supply; b) comprise an underpinning nutrient profile model that considers the overall nutrition	**Nutrient-Specific Labels**	• **Numerical labels** (e.g., NutrInform Battery). Non-interpretative (non evaluative) label, providing numerical information on the content of four nutrients (fat, saturates, sugars, salt) and on the energy value, as well as on how much this represents as a percentage of the daily reference intake
		• **Colour-Coded Labels** (e.g., Multiple Traffic Lights). The label provides numerical information on the content of four nutrients (fat, saturates, sugars, salt) and on energy value, as well as on how much this represents as a percentage of the daily reference intake. Colors are used to classify those nutrients as “low (green), “medium” (amber) or ”high” (red)
	**Summary Labels**	• **Endorsement Logos** (e.g., Keyhole logo). The label provides a synthetic appreciation of a product’s overall nutritional value through a positive (endorsement) logo that is applied only to foods that comply with nutritional criteria
		• **Graded Indicators** (e.g., Nutri-Score) The label provides a synthetic appreciation of a product’s overall nutritional value through a “graded indicator” that provides graded information on the nutritional quality of foods that is applied on all food products

**Table 2:** Prior research on subjective and objective understanding.

	Author	Tested Country	Period of Time
**Objective Understanding**	Ducrot et al.	France	2015
	Egnel et al.	Multiple countries (Denamrk, France, Germany, Spain, UK, United States)	2018
	Egnel et al.	Netherlands	2018
	Egnel et al.	Belgium, Bulgaria, Denmaark, France, Germany, Italy, Netherlands, Poland, Portougal, Spain, Switzerland, United Kingdom)	2020
**Subjective Understanding**	Mazzù et al.	Italy	2020
	Mazzù et al.	Italy, France, Germany, Spain, Portougal, Greece, Romania	2021
	Mazzù et al.	Poland	2021

**Table 3:** Details of sample size by socio-demographic information.

Variables	Slovenia	The Netherlands
**Age**		
18–24	42.9%	8.5%
25–34	42.8%	41.9%
35–44	12.3%	36.7%
45–54	2%	12.1%
55+	0%	0.8%
**Education**		
Lower than diploma	4%	3.6%
Diploma	55%	27%
Bachelor Degree	15%	27.8%
Master Degree	18%	34.7%
PhD	8%	6.9%
**Gender**		
Male	60%	50%
Female	40%	50%
**Income**		
<20k	74%	23%
20–40k	24.4%	38.7%
41–60k	1%	23.4%
61–80k	0%	9.3%
81–100k	0%	4.4%
>100k	0.6%	1.2%
**Profession**		
Student	40%	0.8%
Unemployed	17%	13.7%
Part-time	5%	21%
Full-time	31%	50.4%
Self-employed	6%	10.5%
Housewife	1%	1.6%
Cannot work	0%	2%

**Table 4:** Tested Scales.

	Scale Item	Reference
Comprehensibility		Moser et al. 2010
	I feel well informed by the food label	
	This label is believable and trustworthy	
	This label is easy to interpret	
Help-to-shop		Moser et al. 2010
	This label helps me to understand the product composition	
	This label helps me to understand different nutritional values	
	This label makes it easier to chose food	
Complexity		Moser et al. 2010
	The food label is rather extensive	
	Using this food label to choose food is better than just relying on my own knowledge about what is in them	
Liking		Allen and Janiszewski, 1989
	How do you evaluate the label	
	Bad/Good	
	Unfavourable/Favourable	
	Negative/Positive	

**Table 5:** Scales reliability (Cronbach’s alpha) by country.

	The Netherlands	Slovenia
Comprehensibility	0.71	0.81
Help-to-Shop	0.81	0.79
Complexity	0.77	0.65
Liking	0.89	0.83

**Table 6:** Mean scores by country (healthy and less healthy) on comprehensibility, help-to-shop, complexity, liking, for Nutri-Score and NutrInform Battery adjusted for age, education, income status.

	**NutrInform Battery**		**Nutri-Score**		**t-Test Statistics**		***p*** **Value**	
	Slovenia	Netherland	Slovenia	Netherland	Slovenia	Netherland	Slovenia	Netherland
**Subjective Understanding**								
Comprehensibility	4.92	4.98	3.27	4.31	t(153) = −7.46	t(248) = −3.80	*p* < 0.001	*p* < 0.001
Help to Shop	5.00	4.41	3.37	2.75	t(153) = −7.21	t(248) = −8.86	*p* < 0.001	*p* < 0.001
Complexity	3.99	3.59	2.52	2.43	t(153) = −6.07	t(248) = –6.43	*p* < 0.001	*p* < 0.001
**Liking**	4.96	4.32	4.50	4.41	t(153) = −2.61	t(248) = −4.17	*p* = 0.01	*p* > 0.05
